# Resection of lesions in the ileum of patients with IgG4-related disease may ameliorate disease progression without steroid administration

**DOI:** 10.1186/s40792-018-0546-9

**Published:** 2018-12-29

**Authors:** Akihiro Watanabe, Takashi Goto, Hitomi Kamo, Ryuji Komine, Naomi Kuroki, Takanobu Sugase, Tsuyoshi Takaya, Rintaro Koga, Hiroshi Hojo, Shoji Taniguchi, Kazuhiko Ibusuki, Kazumi Koga

**Affiliations:** 1 Department of Gastrointestinal and General Surgery, Mitsui Memorial Hospital, Tokyo, Japan; 2 Department of Gastrointestinal and General Surgery, Koga General Hospital, Miyazaki, Japan

**Keywords:** IgG4-related sclerosing disease, Small bowel resection, Steroid

## Abstract

**Background:**

IgG4-related disease (IgG4-RD) is a pathological condition that is characterized by an infiltrate composed of IgG4-positive plasma cells and recently recognized as an immune-mediated condition. It causes tissue throughout the body to become stiff and thickened due to autoimmune reactions that cause fibrosis and scarring. Disease-related changes commonly occur in the salivary glands, bile duct, pancreas, and lungs, but are seldom seen in the small bowel. A diagnosis of IgG4-RD is suspected if a high level of IgG4 is found on a blood test. The ideal diagnostic method is pathological examination, but because the clinical manifestations of IgG4-RD are very diverse and nonspecific, the disease may often go undiagnosed until an unrelated biopsy or resection specimen is obtained. The most common treatment for IgG4-RD is steroid use. However, tapering or stopping steroid administration is seen to result in recurrence in approximately 50% of cases. A complete cure is therefore considered extremely difficult.

**Case presentation:**

A 69-year-old man with gastrointestinal obstruction underwent small bowel resection for two lesions. On histopathological examination, the specimen showed features of IgG4-RD. We performed several tests to detect other characteristics of IgG4-RD, but were unable to find any. The patient is being followed up regularly for a year and is being observed for any symptoms of recurrence.

**Conclusions:**

We present a case of IgG4-RD wherein the ileum wall was significantly sclerosed, leading to gastrointestinal tract obstruction; therefore, we resected two sections of the ileum. We believe that resection of IgG4-RD lesions can help avoid long-term steroid use in patients, because the surgery completely eliminates the pathological origins of the condition.

## Background

IgG4-related disease (IgG4-RD) is a pathological condition that is characterized by an infiltrate composed of IgG4-positive plasma cells and recently recognized as an immune-mediated condition that can affect almost any organ [1]. Usually, we suspect that it is present if a high level of IgG4 is found on a blood test. The primary method of diagnosis is pathological examination. However, the clinical manifestations of IgG4-RD are diverse and nonspecific; therefore, the disease may remain undiagnosed until an incidental biopsy or resection specimen is obtained [2].Very little is known regarding the mechanisms and processes involved in the development of this condition; however, type 2 T-helper cells (Th2), which regulate T cell cytokines and B cell activating factor, have been suggested to be associated with the development of this disease [3].

We present a case of IgG4-RD wherein the ileum wall was significantly sclerosed, leading to gastrointestinal tract obstruction, due to which we resected two sections of the ileum.

### Case presentation

The patient was a 69-year-old man who was a known case of type 2 diabetes mellitus (T2DM). He initially presented with sigmoid colon cancer that progressed to obstructed necrotizing enteritis. He had undergone subtotal colectomy (from the ascending colon to the sigmoid colon) in our department. We had created a stoma at the ascending colon and had closed it 1 year later. During this phase, the patient’s recovery was hampered by nontuberculous mycobacterial infection, for which he underwent treatment with isoniazid, rifampicin, and ethambutol at another hospital; he recovered completely from the infection.

He subsequently presented with repeated episodes of gastrointestinal tract obstruction, even after two surgeries. He was then admitted in our department to receive conservative treatment.

Two years after the first surgery, his central venous port was infected, for which he underwent treatment with antibiotics (sulbactam/ampicillin, vancomycin) at another hospital. Subsequently, he presented with obstruction of the gastrointestinal tract again. We therefore attempted to try and resolve this problem.

**Fig. 1:**
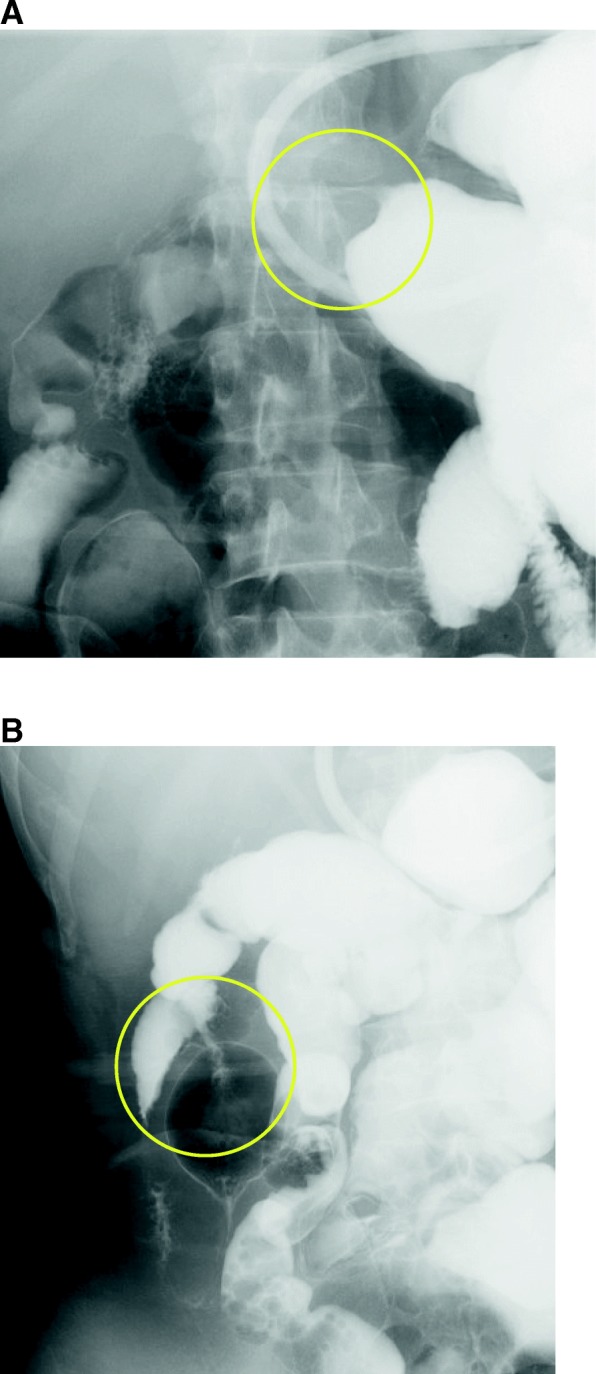
We introduced 200 mL gastrografin into the gastrointestinal tract via a gastric tube. This examination revealed two stenosing sections of the small bowel, as seen on radiological enteroclysis (yellow circles in the image **a** and **b**)

**Fig. 2:**
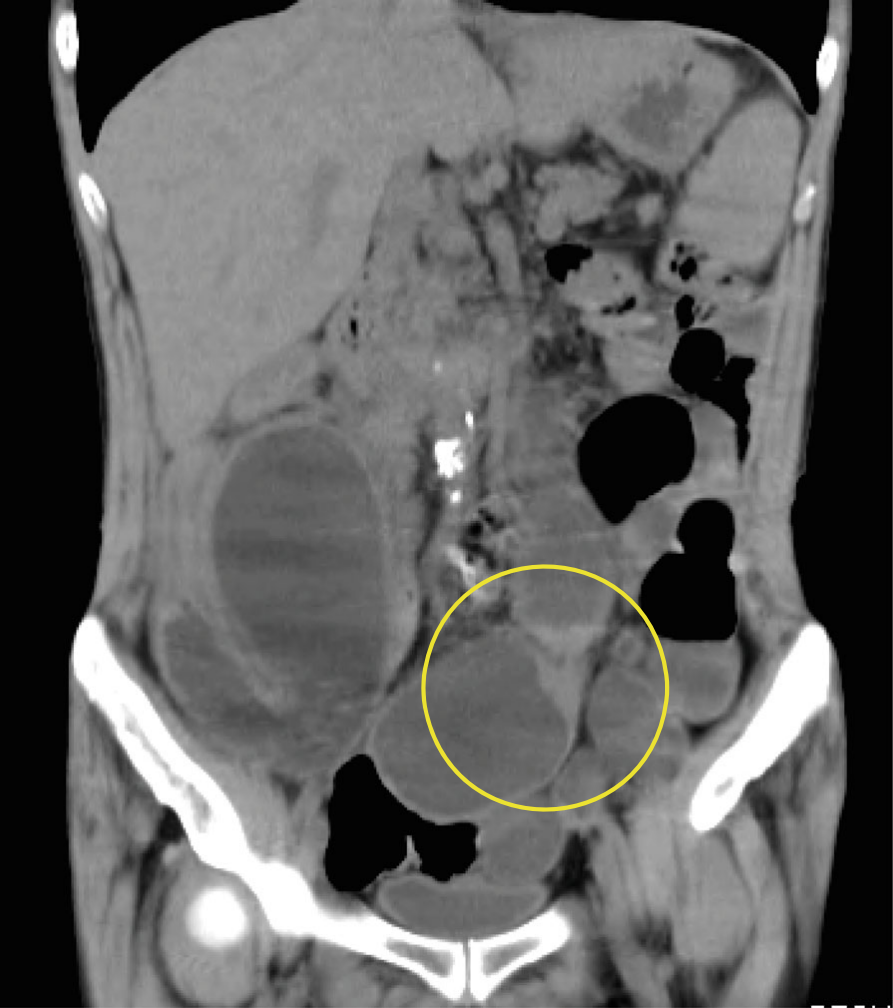
The wall of the small bowel is thick and stenosed, as seen on computed tomography (yellow circle)

Blood tests showed renal dysfunction (serum creatinine level 1.18 mg/dL), anemia (hemoglobin 9.0 g/dL), and a slightly elevated CA19-9 level (46 U/mL). Radiological enteroclysis revealed two stenosed sections of the small bowel (Fig. 1). Computed tomography of the abdomen also indicated that the wall of the small bowel was thick and stenosed (Fig. 2).

We continued conservative treatment (using an ileus tube), but his condition did not improve. We then decided to operate to resolve this condition.

**Fig. 3:**
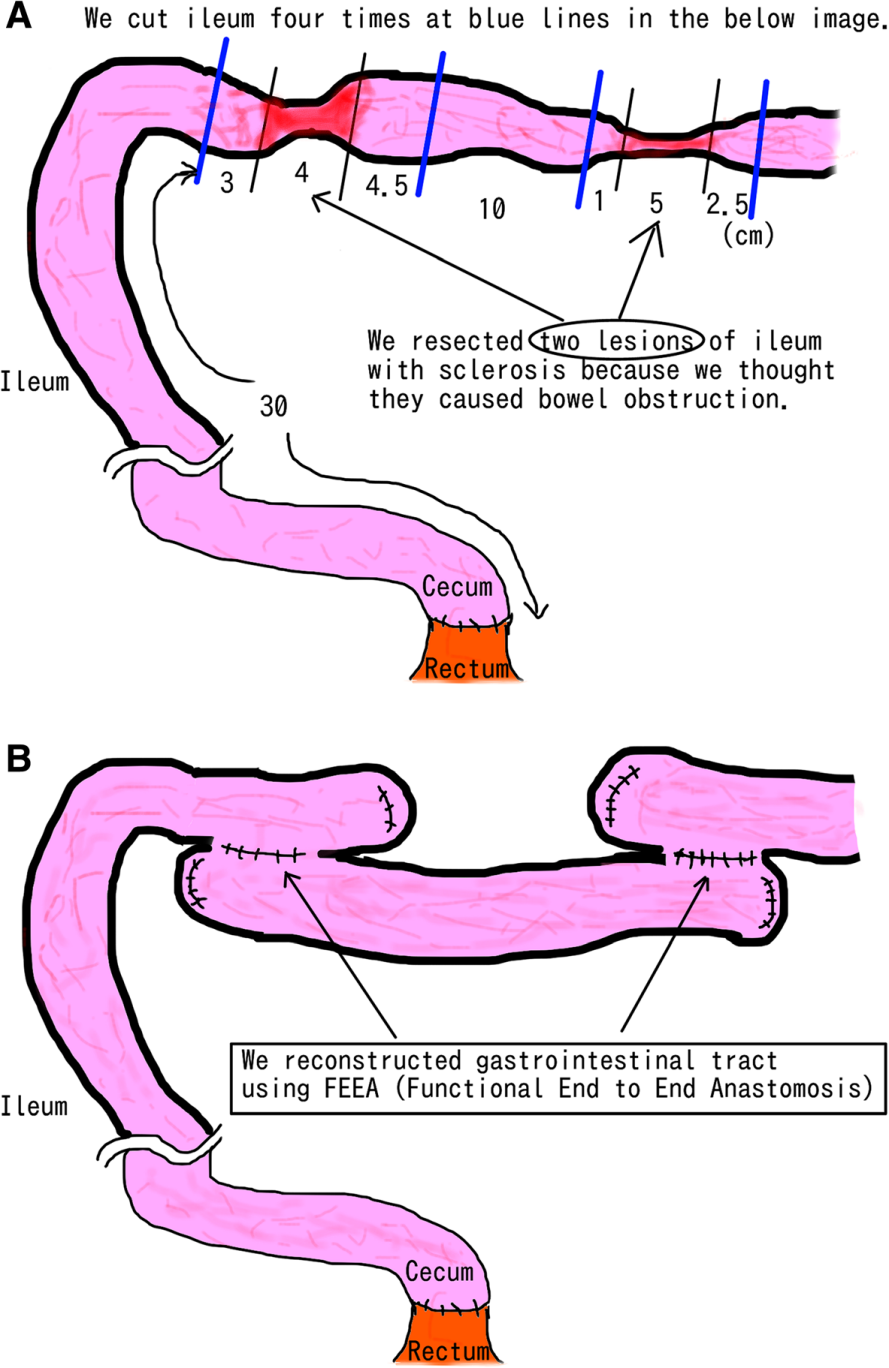
The patient’s cecum and rectum were anastomosed due to the previous surgery (subtotal colectomy). We recognized two sections of small bowel 33 cm and 53 cm from the terminal ileum seemed to be sclerotic, with extraordinarily thick walls (**a**). We resected these two lesions and anastomosed them using FEEA (functional end-to-end anastomosis), thus obtaining two specimens (**b**)

**Fig. 4:**
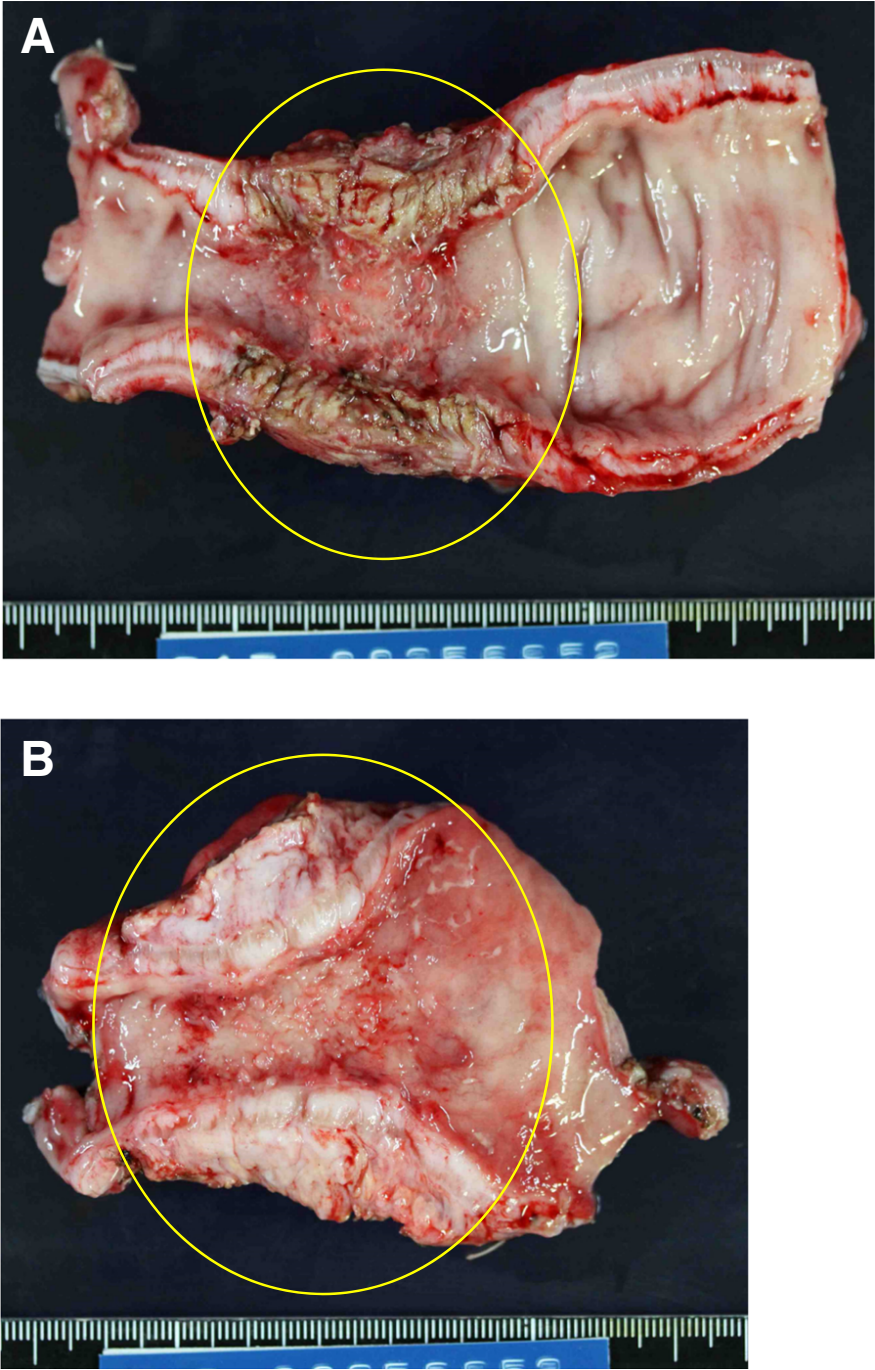
Images a and b are macroscopic findings of the specimen. Two specimens of the ileum with a rubbery appearance and extraordinarily thick walls (yellow circles in the image **a** and **b**)

In the surgery, we recognized two sections of small bowel 30 cm and 53 cm from the terminal ileum seemed to be fibrosed, with extraordinarily thick walls. We resected these sections with a few centimeters margin and anastomosed the remaining bowel using functional end-to-end anastomosis, finally obtaining two specimens (Figs. 3 and 4). We also performed tube splinting of the small bowel to prevent further gastrointestinal tract obstruction.

**Fig. 5:**
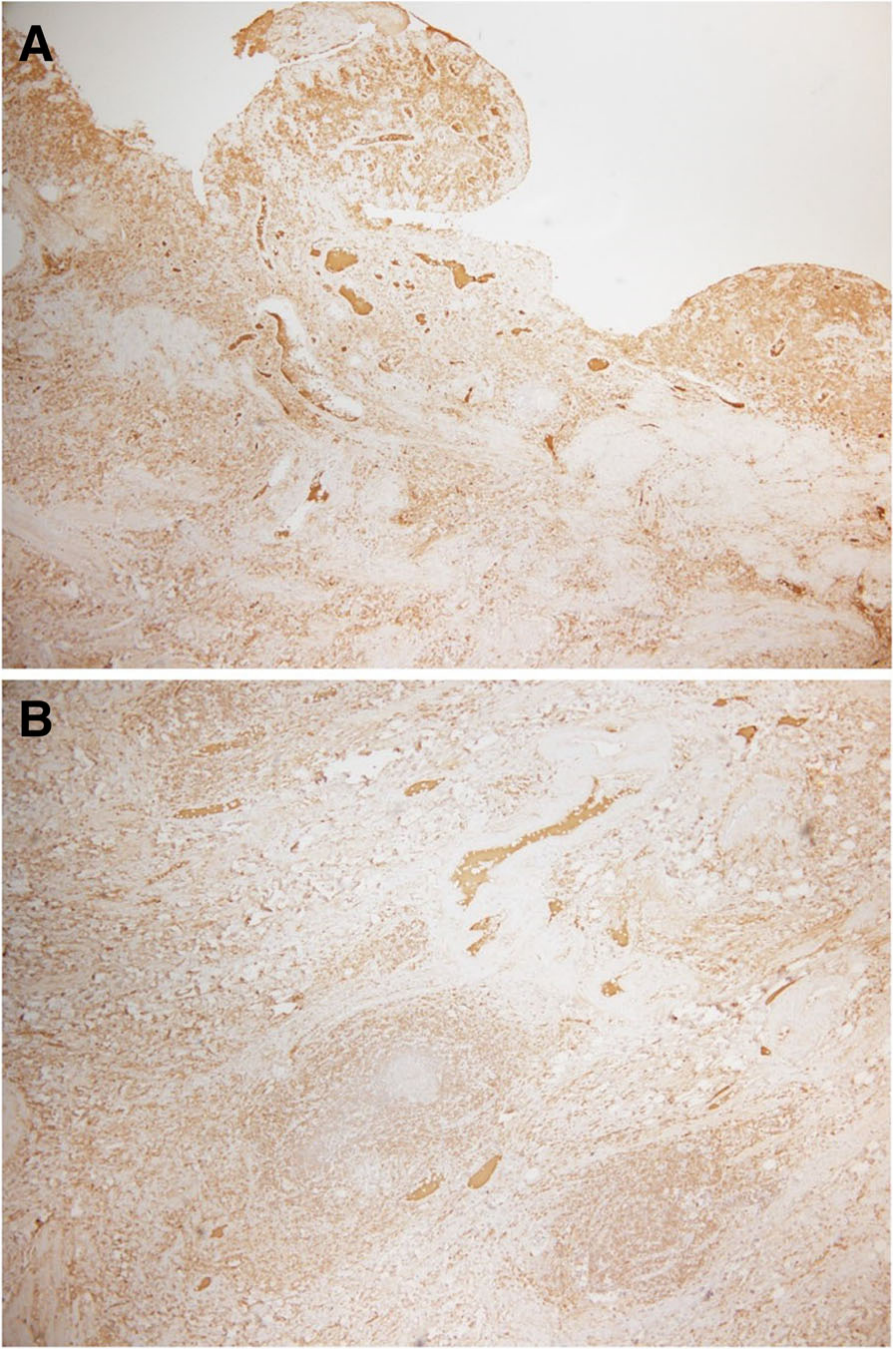
Images **a** and **b** are histological findings of the specimen. A large number of plasma cells and IgG4-positive cells are seen in the specimen. The ratio of IgG4-positive cells to non IgG-positive cells is 87%

After the surgery, pathological examination yielded a large number of plasma cells and IgG4-positive cells in the specimen; the ratio of IgG4-positive cells to non-IgG-positive cells was 87% (350/404). We then realized that the specimen showed features of IgG4-RD (Fig. 5). Based on the diagnostic criteria, this case was classified into the probable diagnosis group.

We then conducted some tests to further clarify the diagnosis of IgG4-related disease. The blood IgG4 level was found to be 52.0 mg/dL, which is within the normal limit. We performed magnetic resonance cholangiopancreatography (MRCP) to detect signs of IgG4-RD in the pancreas and bile duct, but did not find any. We therefore concluded that there were no findings that suggested IgG4-RD, except for in the small bowel.

The patient was discharged to his home on the 39th postoperative day. He has been following up regularly for a year with us and has not shown any symptom of recurrence of IgG4-RD.

## Discussion

The primary pathological features of IgG4-RD are swelling around the internal organs, thickened lesions due to infiltration by lymphocytes and IgG4-positive plasma cells, and fibrosis of tissue throughout the body [4].

IgG4-RD rarely affects the small bowel. Several studies describe the common primary locations of the lesions as the salivary glands, bile duct, pancreas, and lung; however, none of these studies describe any cases with lesions in the small bowel [5].

The diagnostic criteria of IgG4-RD are quite clearly defined [6]. There are three main items in the list. Patients who fulfill all the criteria are classified into the definite diagnostic group, while patients who fulfill criteria 1 and 3 are classified into the probable diagnostic group. The criteria are:

1. Characteristic diffuse or segmental swollen, massive, nodule, or hypertrophic lesions in single or multiple organs
2. Elevated serum IgG4 levels (above 135 mg/dL)
3. Overwhelming infiltration of lymphocytes and plasma cells and sclerosing and infiltration of IgG4-positive plasma cells; the ratio of IgG4/IgG is over 40%, with over 10 IgG4-positive plasma cells seen per HPF.

The etiology of IgG4-RD seems to be autoimmune, based on the presence of high serum IgG4 levels and infiltration of IgG4-positive plasma cells. The disease is associated with both Th2 and regulatory T cell (Treg) immune responses. Th2 responses are also linked to the development of allergies and bronchial asthma, both of which are conditions that are more prevalent in patients with IgG4-RD [2], but the detailed etiopathology is still unknown [7].

The most commonly used and suitable medication used to treat IgG4-RD is steroids [8, 9]. Kamisawa et al. reported that oral prednisolone is usually started at 0.6 mg/kg/day; for the maintenance, the steroid dose is then tapered by 5 mg every 1 to 2 weeks [10]. The ideal time frame of prednisolone administration is 1 to 2 years, which can be paused in case of remission. However, the treatment in case of recurrence has not been established. Steroids are effective in treating nearly 100% of cases, but approximately 50% cases are seen to relapse if steroids are reduced or stopped. Therefore, a total cure should not be expected [9, 11, 12].

This is a very unusual case of IgG4-RD wherein the patient presentation was different, and the signs of the condition were not present in any of the usual locations. Additionally, symptoms of recurrence have not developed even a year after surgery. In this case, the serum IgG4 level was found to be within normal limits. However, we believe that this was only because we checked the IgG4 levels after surgery. This possibly occurred because we resected the lesions that were likely producing the IgG4-positive plasma cells. This suggests that resection of the primary lesion in cases of IgG4-RD decreases the IgG4 level. This hypothesis is also supported by a report by Fujita et al., of a case of IgG4-RD treated using steroids. In this case, prednisolone was used, but resection was not performed. The patient’s symptoms improved, but the number of IgG4-positive cells did not change after remission [13].

In our case, we have determined that if symptoms of recurrence are noted, the best course to follow would be steroid treatment with careful monitoring, keeping in mind the patient’s T2DM and latent nontuberculous mycobacterial infection, because the utility of re-operation is yet unknown.

This patient underwent subtotal colectomy for obstructed necrotizing enteritis by sigmoid colon cancer, but it is not related to IgG4-RD. This patient developed gastrointestinal obstruction only because of sclerosis and thickening of the ileum by etiology of IgG4-RD. Resecting the lesion seemed to solve the problem, but recurrence is possible in the future. Therefore, his condition needs to be strictly monitored during follow-ups.

We suggest that operation is one of the feasible tactics of treatment of IgG4-RD in a particular situation, such as in a case we are reluctant to use steroid (e.g., a patient with diabetes mellitus). Xiao et al. reported that among 39 cases of IgG4-related cholangitis, 4 underwent surgery [13]. Among these 4 cases, 2 underwent pancreatoduodenectomy, 1 underwent choledochojejunostomy, and the fourth one underwent choledochoplasty. Over a follow-up period of 9 to 36 months, all patients achieved resolution of stricture of the bile duct and normalization of liver enzyme levels. Other authors reported cases underwent surgery in several areas for example mesentery and lung for IgG4-RD patients and did not recur within follow-up period [14, 15]. These reports suggest that surgical intervention could be more effective than using steroids to control IgG4-RD.

## Conclusion

Experience of additional cases is needed to determine the symptoms and characteristics of IgG4-RD with sclerosing changes in the small bowel, as very few cases have been reported so far. Resection of the lesions of IgG4-RD could be considered to avoid long-term steroid use in patients.

## Abbreviations

IgG4-RD: IgG4-related disease
MRCP: Magnetic resonance cholangiopancreatography
T2: Type 2 T-helper cell
T2DM: Type 2 diabetes mellitus
Treg: Regulatory T cell
